# A Pilot Study Assessing the Oral Microbiome in Women of Menopausal Age: Do Oral Nitrate–Reducing Bacteria Play a Role?

**DOI:** 10.1016/j.identj.2026.109518

**Published:** 2026-03-25

**Authors:** Katie J. Muddiman, Amazon Doble, Abish S. Stephen, Raul Bescos, Charlotte S. Illsley, Tomas L. Nicholas, Sally Hanks, Lisa du Toit, Zoë L.S. Brookes

**Affiliations:** a School of Biomedical Science, University of Plymouth, Plymouth, UK; b Peninsula Dental School, University of Plymouth, Plymouth, UK; c Queen Mary Dental School, Queen Mary University of London, London, UK; d School of Health Professions, University of Plymouth, Plymouth, UK

**Keywords:** Menopause, Dentistry, Oral microbiome, Red complex bacteria, Periodontal

## Abstract

**Introduction:**

The links between oral health and female ageing are poorly understood, but many changes occur in the oral cavity of menopausal women that affect quality of life, and few current oral health interventions consider gender as part of their approach. The aim of this pilot study was to test the hypothesis that the oral microbiome and microenvironment change during female ageing and are thus worthy of further consideration both experimentally and clinically.

**Methods:**

This observational pilot study retrospectively assessed women aged 18 to 89 years (n = 60) attending a UK primary care dental school facility for blood pressure screening, further analysing the salivary oral microbiome using metagenomics and the biochemical microenvironment using high-performance liquid chromatography. Periodontal health screening (Basic Periodontal Examination [BPE]) was then conducted as part of routine clinical care.

**Results:**

The cross-sectional design classified women into <32 years (n = 18), 40 to 49 years (n = 10), 50 to 59 years (n = 20), and 60+ years (n = 12), but the differences in salivary oestradiol levels between groups were inconclusive. Small numbers were not enough to detect differences in oral microbiome abundance, but nitrate-reducing species (*P* < .05), nitrate-nitrite–reducing activity (*P* < .05), and buffering capacity all increased as women aged 60+ years (*P* < .01), warranting increased numbers. Ageing women also had higher blood pressure (*P* > .05), were more likely to have periodontal pockets >5.5 mm (BPE4), and had an increased abundance of *Porphyromonas* (*P* < .05), but a full periodontal assessment is needed.

**Conclusions:**

These observations suggest that the composition of the oral microbiome changes as women age, and thus, prospective and longitudinal oral microbiome studies with larger numbers are needed, including concurrent full periodontal assessment, plasma hormonal levels, and salivary flow. However, this study suggests that the oral microbiome in older women may require special consideration, with an increased focus on tailored oral hygiene interventions for this group.

## Introduction

Female ageing is accompanied by profound physiological changes,^1^ many of which are influenced by the decline in sex hormones such as oestrogen and progesterone.^2^ As women age, one of the most significant transitions is menopause, defined as the permanent cessation of menstruation resulting from the loss of ovarian follicular activity.^3^ This typically occurs at an average age of 45 to 56 years^2^ and is preceded by the perimenopausal period, often beginning in the mid-40s, during which women also experience a gradual decline in circulating hormone levels and various systemic changes.^4^ Over 70% of age-related diseases are influenced by the effects of reproductive ageing^5^; thus, no study of women ageing would be complete without integrating this consideration.

Cardiovascular health is significantly impacted as women age,^6^ and it is arguably underinvestigated specifically in women, in whom mechanisms of disease may be different from those of males. Declining oestrogen levels, for example, can contribute to increased blood pressure and vascular stiffness observed in this population with age,^7^ as oestrogen is able to trigger vasodilation, allowing blood to flow through arteries more easily.^8^ Although hormone replacement therapy (HRT) has proven effective in managing many menopausal symptoms, including protective effects on cardiovascular health,^9^ as well as regulation of blood vessel tone and bone density loss,^1^ the effects of female ageing on oral health remain relatively underexplored.^10^ Assumed to result from hormonal changes, many women in the menopausal and perimenopausal age groups report a change from their previously experienced oral physiology, with symptoms such as dry mouth,^11^ burning mouth syndrome, and taste disturbances^12^ developing, which can have a considerable impact on quality of life.

It has also been reported that the composition and function of the bacterial communities in the mouth, the oral microbiome, change as women age.^13^ Other studies have reported, in contrast, that the salivary microbiome remains stable during menopause.^14^ It may be that any changes in bacteria are secondary to, and dependent on, individual-specific hormonal changes that result in an altered biochemical environment and “drier” mouth as women age,^14^ alongside other factors such as diet, exercise, and medications that can also affect oral health. However, the exact biochemical changes and bacterial species affected during menopause remain undefined thus far.^13^^,^^15^ Whilst this subject attracts review and current calls for more research, there remain few original research studies in this area considering gender, which is important, as a dysbiotic oral microbiome with a predominance of pathogenic bacteria could be the reason for the increased risk of dental decay (caries) and gum (periodontal) disease in older women.^13^^,^^16^^,^^17^

It is also known that the composition of the oral microbiome affects cardiovascular health,^18^^,^^19^ including blood pressure, and thus it is important to ensure that older women maintain a healthy and diverse oral microbiome to help reduce the risk factors for heart attacks and strokes. It is further important to understand the oral microbiome specific to females, so that clinical interventions used to manage oral disease and symptoms are appropriate to gender. Ensuring this starts by understanding whether, or rather how, a woman’s oral microbiome changes over time.

Of particular interest within the oral microbiome are the nitrate-reducing bacteria, which play a key role in the nitrate-nitrite–nitric oxide (NO) pathway that helps regulate vascular function and blood pressure.^18^^,^^20^, ^21^, ^22^, ^23^ Bacteria such as *Veillonella, Rothia, Neisseria, Actinomyces*, and, to a lesser extent, *Prevotella* genera are part of a healthy oral microbiome.^24^ Such oral nitrate–reducing bacteria (ONRB) facilitate the conversion of dietary nitrate into nitrite, which, once ingested, can form NO that dilates blood vessels to lower blood pressure through homeostatic mechanisms.^25^ Interestingly, *Prevotella copri* increases in abundance and *Veillonella tobetsuensis* decreases after menopause,^14^ but the abundance and activity of all nitrate-reducing species remain underexplored in women as they age. As mentioned, ageing women also tend to exhibit increasing blood pressure^26^; thus, as oral nitrate–reducing species mechanistically regulate blood pressure, it is important to investigate changes in these oral nitrate–reducing species in combination with an assessment of systemic health.

The primary aim of this study therefore was to test the hypothesis that the oral microbiome and microenvironment change during female ageing, looking in more detail at nitrate-reducing bacteria, to determine whether the oral microbiome is worthy of further consideration both experimentally and clinically during menopause. It also aimed to determine whether periodontal disease and blood pressure concurrently increased, as suggested by previous studies, to begin interpretating findings in the context of oral and systemic health. This information was important to elucidate ahead of larger studies and ultimately before suggesting gender-specific clinical interventions to potentially modulate the oral microbiome and improve oral health in older women.

## Materials and methods

### Ethics

The samples used in this observational study were approved by the Human Ethics Committee of the University of Plymouth (#2684 and #16/17–666) between 2020 and 2023 for a different study involving blood pressure screening, but with consent for secondary analysis of stored samples and participant data.^27^ The study began in 2020 as a nurse-led clinical blood pressure screening service within our dental social enterprise setting,^27^ which subsequently acquired an ethics amendment and Human Tissue Act (HTA) approval to collect saliva from participants to concurrently measure the oral microbiome. The blood pressure screening did not provide interventions beyond routine care, and the data reported here relate to a secondary analysis of oral microbiome samples from pre- and postmenopausal women undergoing blood pressure screening. Hence, being retrospective and observational in nature, this was not an interventional or randomised controlled trial that could be registered under a public clinical trials registry (eg, www.clinicaltrials.gov) as part of usual good clinical practice. Nevertheless, the protocol and design, including data recording, complied with all ethical standards outlined by the responsible committee on human research experimentation, including safe sampling and storage of tissues in accordance with the University of Plymouth Ethics Committee processes on HTA approval, patient confidentiality, data collection, and storage in compliance with General Data Protection Regulation (GDPR).

### Inclusion and exclusion

**Figure 1:**
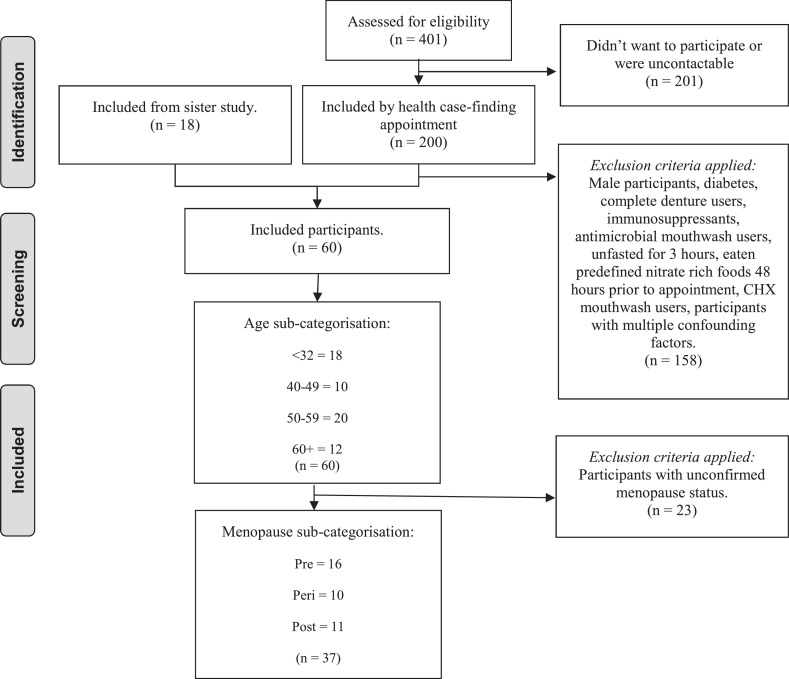
Inclusion criteria for including and categorising n = 60 women in the “women ageing” and “menopausal ageing” groups for later analysis of the salivary oral microbiome and salivary biochemical markers.

Only female participants were included in the analysis (n = 60), aged 18 to 89 years. Inclusion criteria are outlined in Figure 1, with biological sex status also confirmed. At the time of sample collection, the confounding factors that were excluded *preexperimentally* included diagnosis of diabetes (type 1 and 2), complete denture users, taking immunosuppressants, prehypertension result, antimicrobial mouthwash use within the previous 3 months, unfasted for 3 hours, and having eaten predefined nitrate-rich foods 48 hours prior to the appointment.^28^^,^^29^ Participants taking HRT or contraceptives were not excluded from group numbers as these data were not collected robustly.

A multiple linear regression analysis was also used *postexperimentally* to determine the inclusion and exclusion of participants (see statistics section). The factors assessed for their impact on oral microbial Shannon diversity included smoking, vaping, periodontal health, any antibiotic use, any mouthwash use, and multiple confounders. Mouthwash (*t* = –2.30, *P* = .020) use and multiple confounders (ie, 2 or more of the confounders listed above) (*t* = –2.69, *P* = .016) were statistically significant predictors of oral microbial Shannon diversity in our population, and thus participants using mouthwash and participants with multiple confounding factors were excluded from all our experimental group numbers and the oral microbiome data presented henceforth. Periodontal health (see oral health section) was included, as the literature links this to ageing, and the aim of this study was to identify the possible interplay between periodontal health and the oral microbiome for further investigation.

### Experimental groups

Participants were categorised into the following “women ageing groups” to assess changes over time: (1) <32 years (n = 18), (2) 40 to 49 years (n = 10), (3) 50 to 59 years (n = 20), and (4) 60+ years (n = 12). Participants were also assigned into a second group based on the literature,^30^ being one of the following “menopausal groups” related to age where menopause was assumed based on a mean menopausal age of 49 years in the UK and an interquartile range of 45 to 51 years, with women unlikely to be menstruating in any form after 60 years of age or likely to be to menstruating at less than 32 years unless a medical condition was identified^30^: (1) 32 years and younger (ie, premenopause [Pre], n = 16), (2) 45 to 57 years who were known to be menstruating in some capacity (ie, perimenopause [Peri], n = 10), and (3) 60+ years who were known to have stopped menstruating (ie, postmenopause [Post], n = 10). This entailed excluding women whose menstruation status was unclear or was not disclosed, as whilst age may correlate with menopause, there can be variations and exceptions due to early or late menopause in some women.

### Data collection

#### Cardiovascular health

Blood pressure and body mass index (BMI) were first collected using the protocol published by Doble et al.^27^ Sociodemographic information, including smoking status, was retrieved from the medical history questionnaires.^27^^,^^31^

#### Oral sample collection

An unstimulated saliva sample was collected over 5 minutes and then subject to centrifugation (14,000 rpm, 4 °C). The pellet and supernatant were separated and stored at –80 °C. After saliva collection, a sodium nitrate rinse (80 μmol, in water) was held in the mouth (like a mouthwash) for 5 minutes for later analysis of the nitrate-reducing activity within the mouth.^28^^,^^32^ Batch analysis was undertaken at the end of the collection period.

#### Salivary biochemical analysis

Salivary supernatant was analysed for quantification of the biochemical markers nitrate, nitrite, ammonia, pH, buffering capacity, lactate, glucose, IL-6, and IL-10.^33^, ^34^

#### Salivary oral microbiome analysis

DNA from stored saliva pellets was extracted using the DNeasy PowerSoil Pro Kit (QIAGEN), quantified on a NanoDrop 2000 Spectrophotometer (Thermo Scientific), and sent to Queen Mary University London Genome Centre. Amplification of the 16S rRNA V1-2 region was carried out using universal 16S primers 27F YM (5-AGAGTTTGATYMTGGCT CAG, where Y is C or T)^35^ and 338R (5′-TGCTGCCTCCCGTAGRAGT-3′).^36^ The libraries were sequenced using the multiplexed, barcoded 250-bp paired-end read methodology using the NextSeq2000 platform (Illumina) according to the manufacturer’s instructions. Samples were demultiplexed and then exported as fastq files.

Bioinformatic analyses were performed on the fastq files using RStudio (V2024.04.0) to remove primers and trim sequences. Low-quality ends of the forward and reverse reads were trimmed, and the reads were filtered using default parameters (maxN=0; maxEE=2, 4). The filtered reads were then error corrected and merged, and chimeras were removed using the DADA2 pipeline (V1.16)^37^ (DADA2, 2024). Taxonomy was assigned to the error-corrected amplicon sequence variants (ASVs) using the eHOMD database’s 16S rRNA training set.^38^ Sequence counts of ASVs that mapped to Human Microbial Taxa at the genus, species, or subspecies levels were agglomerated in phyloseq*.* The read depth was approximately 0.8365 million reads (±87,041.5).^37^

#### Oral reducing bacteria activity and abundance

Within the oral microbiome analysis, the combined abundance of all oral nitrate–reducing species is referred to as ONRB. These bacteria have been classified as nitrate reducers, as previously confirmed in the literature.^23^^,^^39^ The bacteria defined under this classification were then measured for their combined oral nitrate–reducing activity (ONRA) using high-performance liquid chromatography analysis of the nitrate rinse, as previously described by our laboratory.^28^^,^^32^

#### Oral health analysis

Periodontal health was measured separately from oral sample collection, as these participants were also patients within student dental clinics at the University of Plymouth. Ethical approval was gained to acquire medical and dental histories from their dental records. For this study, participants with a Basic Periodontal Examination (BPE) score of 4 in at least 1 sextant were defined as “potential periodontal concern,” and according to British Society of Periodontology (BSP) guidelines,^40^ a BPE score of 4 correlates with a probing depth of >5.5 mm, and thus, whilst not diagnostic, it can be a good indicator of “potential periodontal disease.” Retrospectively collected periodontal health data (no more than 6 weeks apart from the blood pressure screening) were available only for the following participants in each women’s ageing group: <32 years (n = 16), 40 to 49 years (n = 6), 50 to 59 years (n = 14), and 60+ years (n = 7). Oral health data were available for the following participants in each menopause group: Pre (n = 16), Peri (n = 6), and Post (n = 7).

### Statistical analysis

Multiple linear regression analyses were carried out using XLSTAT and Excel to evaluate the associations between the dependent and independent variables. Basic data analysis was conducted using Past4 (V4.16) for statistical analysis.^41^ Normal distribution of data was assessed using a Shapiro-Wilk test. Differences between 2 groups were analysed using independent *t* tests (normally distributed) or a Mann-Whitney test (non-normally distributed). Categorical variables were assessed using a χ^2^ test. Biomarker data analysis and graphs were produced in GraphPad Prism 10.3.0 (GraphPad Software). Normal distribution was assessed using a Shapiro-Wilk test. Differences between groups were investigated using a Brown-Forsythe analysis of variance with Dunnett’s T3 multiple comparisons test (normally distributed data) or a Kruskal-Wallis test (non-normally distributed data).

The α- and β-diversity were examined using Past4 (V4.16). The α-diversity profiles were displayed using boxplots of the Shannon diversity indices (7H). Statistical comparison was made using a Mann-Whitney test, with pairwise *P* values being Bonferroni corrected (α). The β-diversity profiles were displayed using nonmetric multidimensional scaling plots of the oral microbiota in different women ageing or menopause groups, using the Bray-Curtis index. Statistical comparison was made using a 1-way permutational multivariate analysis of variance, with pairwise *P* values being Bonferroni corrected (β). Differential abundance analysis of oral bacteria was also carried out using ANCOM-BC (RStudio, V2024.04.0). Significantly differentially abundant taxa between groups were represented using forest plots of log fold changes (RStudio, V2024.04.0).

## Results

### Cardiovascular health

**Figure 2:**
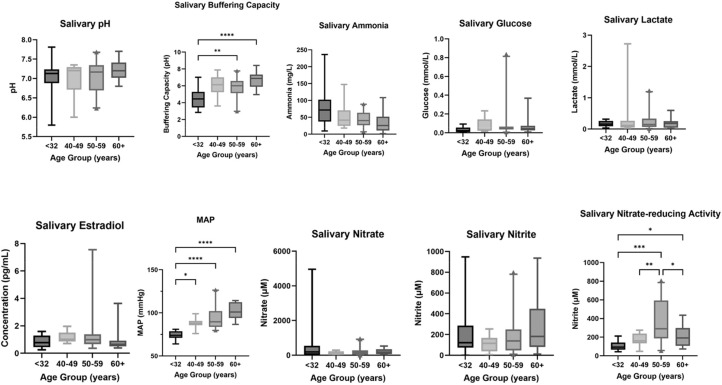
Salivary biomarkers (plus mean arterial pressure) in the women ageing group. Normality of data was assessed using a Shapiro-Wilk test. A Kruskal-Wallis test with Dunn’s multiple comparisons test was used for all, except for salivary buffering capacity, where a Brown-Forsythe analysis of variance with Dunnett’s T3 comparisons test was used instead. Whiskers show the 5th and 95th percentiles. *P < .1, **P < .01, ***P < .001, ****P < .0001 (<32, n = 16; 40-49, n = 10; 50-59, n = 20; 60+, n = 12; except salivary glucose 60+ years [n = 11], salivary nitrate-reducing activity <32 years [n = 15] and 40-49 years [n = 9], and salivary oestradiol <32 years [n = 15] and 40-49 years [n = 9]).

**Figure 3:**
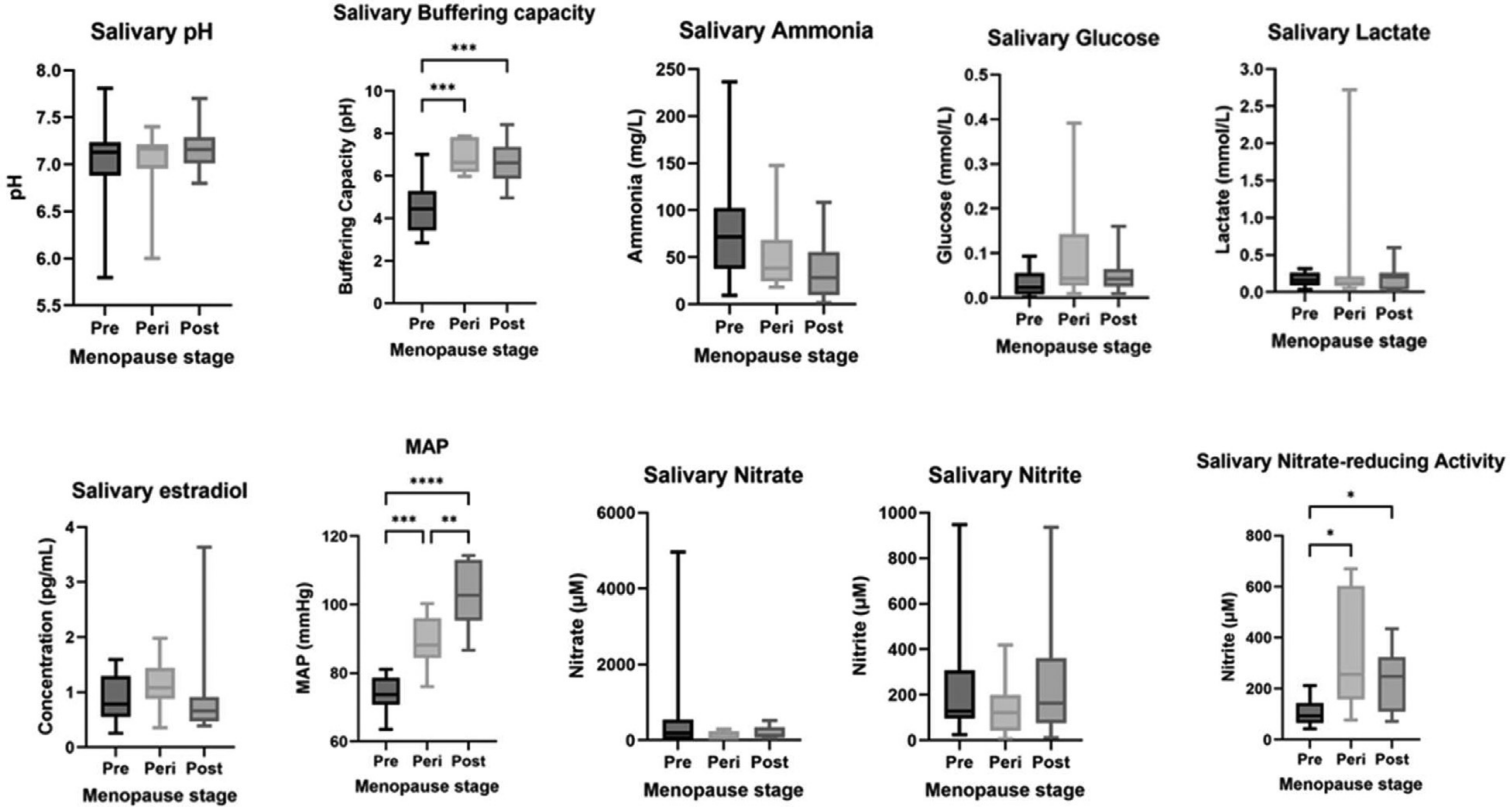
Salivary biomarkers (plus mean arterial pressure [MAP]) in the menopausal ageing group. Normality of data was assessed using a Shapiro-Wilk test. A Kruskal-Wallis test with Dunn’s multiple comparisons test was used for all except MAP and salivary nitrite-reducing activity, where a Brown-Forsythe analysis of variance with Dunnett’s T3 comparisons test was used instead. Whiskers show the 5th and 95th percentiles. *P < .1, **P < .01, ***P < .001, ****P < .0001 (Pre, n = 16; Peri, n = 10; Post, n = 13).

Systolic blood pressure, diastolic blood pressure, mean arterial pressure (MAP), and BMI increased with age in our women ageing classifications, whereas systolic blood pressure and MAP only increased with the menopause classifications (Table 1). MAP was significantly different between the women ageing groups (*P* < .0001), with MAP being lowest in the <32-year age group and highest in the 60+-year age group (Figures 2 and 3). MAP was significantly different between the menopause ageing groups (*P* < .01 to *P* < .001) (Figures 2 and 3), with MAP being lowest in the premenopause group and highest in the postmenopause group.

**Table 1:** Population characteristics.

Characteristic	Female ageing				Menopause ageing		
	<32 years (n = 16)	40-49 years (n = 10)	50-59 years (n = 20)	60+ years (n = 12)	Pre (n = 16)	Peri (n = 10)	Post (n = 11)
Age, y	22.7 ± 0.9	43.9 ± 0.7	54.1 ± 0.5	69.9 ± 2.1	22.75 ± 0.95	49.4 ± 1.78	69.8 ± 2.3
Systolic BP, mm Hg	100.0 ± 1.6	115.9 ± 2.7	125.3 ± 4.3	141.2 ± 5.4	100.28 ± 1.66	119.7 ± 2.99	142.18 ± 19.37
Diastolic BP, mm Hg	60.8 ± 1.1	74.0 ± 1.6	74.7 ± 2.5	82.0 ± 1.8	100.28 ± 1.66	74.2 ± 2.26	82.6 ± 1.83
MAP, mm Hg	74.0 ± 1.2	87.9 ± 1.8	94.1 ± 3.0	101.7 ± 2.8	74.02 ± 1.23	89.37 ± 2.35	102.48 ± 2.94
BMI, kg/m^2^	21.6 ± 0.5	27.6 ± 2.18	29.0 ± 1.5	29.6 ± 8.5	21.6 ± 0.5	30.8 ± 2.12	28.8 ± 1.25
Smoking (yes/no), No. (%)	0 (0)	3 (30)	6 (10)	1 (8.3)	0 (0)	2 (20)	1 (9)

Abbreviations: BMI, body mass index; BP, blood pressure; MAP, mean arterial pressure.

Values are presented as mean ± SEM or number (%).

### Oral health

No “potential periodontal disease” (BPE 4) was seen in women aged <32 years, but “potential periodontal disease” was identified in women aged 40 to 49 years (n = 2), 50 to 59 years (n = 1), and >60 years (n = 3). No potential periodontal disease was seen in the premenopausal women, but potential periodontal disease was identified in the perimenopausal age group (n = 1) and postmenopausal age group (n = 3).

### Salivary biochemical analysis

*Women ageing:* Salivary buffering capacity was significantly different between some age groups (*P* < .01 to *P* < .0001; Figure 2), with salivary buffering capacity lowest in women aged <32 years and highest in women >60 years (Figure 3). Salivary ONRA was significantly different between ages (*P* < .01 to *P* < .001), with ONRA being lowest in women aged <32 years and highest in women aged 50 to 59 years. However, salivary pH did not significantly change with women ageing, nor did levels of salivary oestradiol.

*Menopausal ageing:* Salivary buffering capacity was significantly different between the menopause groups (*P* < .001), with salivary buffering capacity being lowest in the premenopause group and highest in the postmenopause group (Figure 3). However, salivary pH did not change with menopausal age, nor did levels of salivary oestradiol.

### Salivary oral microbiome analysis

**Figure 4:**
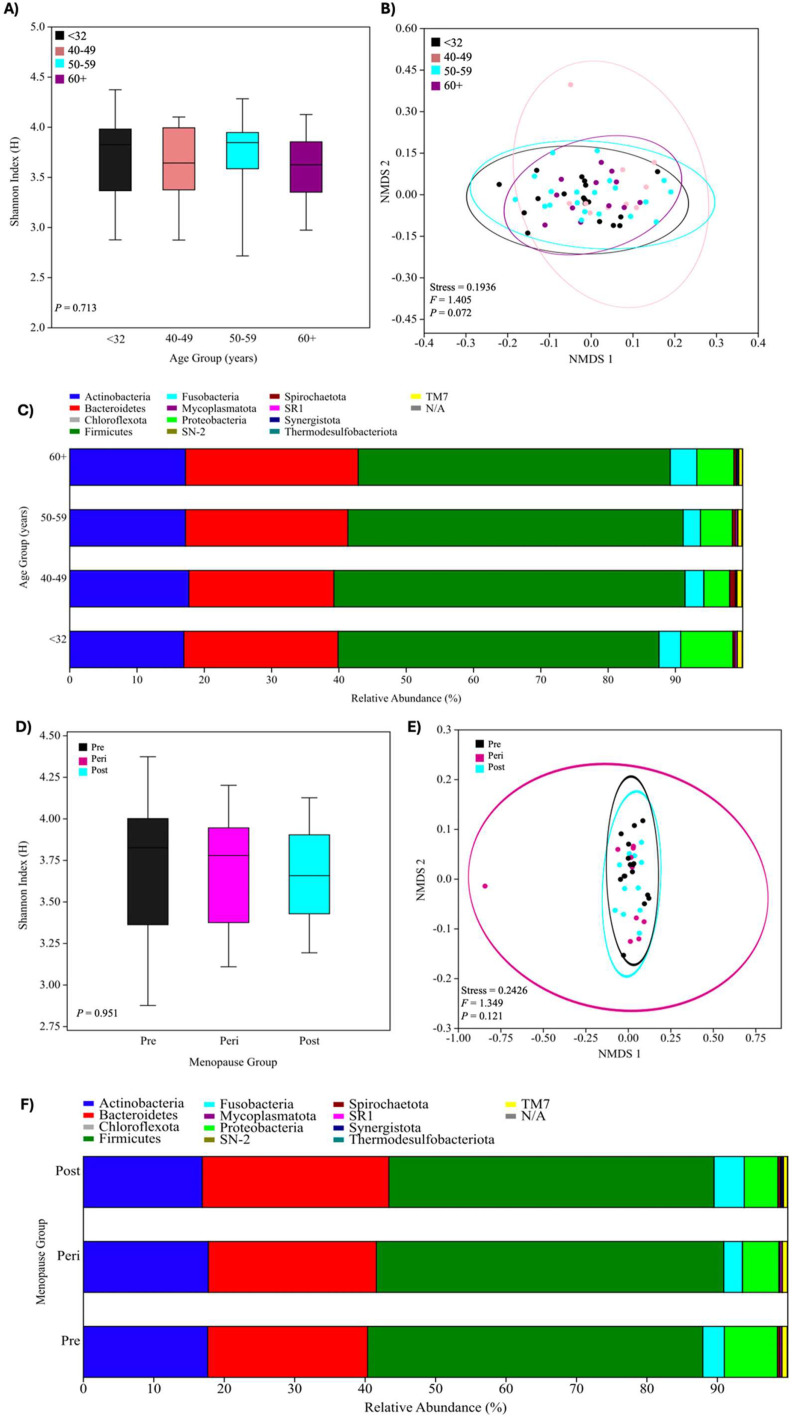
Oral microbiome diversity of composition in the women ageing and menopausal ageing groups. (A) The α-diversity profile, displayed using a boxplot of Shannon diversity indices (H) of oral microbiota. (B) The β-diversity profile, displayed using nonmetric multidimensional scaling (NMDS) plots. Ellipsis represents the 95% confidence intervals for groups. (C) Stacked bar chart displaying the relative abundance of the main bacterial phyla. Phyla are assigned according to the National Institutes of Health (NIH) National Centre for Biotechnology Information (NCBI) taxonomy database. Data were considered statistically significant at *P* < .05, displayed with an asterisk in each plot: *<0.05, **<0.01, and ***<0.001 (<32, n = 18; 40-49, n = 10; 50-59, n = 20; 60+, n = 12). (D) The α-diversity profile, displayed using a boxplot of Shannon diversity indices (H) of oral microbiota. (E) The β-diversity profile, displayed using NMDS plots. Ellipsis represents the 95% confidence intervals for groups. (F) Stacked bar chart displaying the relative abundance of the main bacterial phyla. Phyla are assigned according to the NIH NCBA taxonomy database. Data were considered statistically significant at *P* < .05, displayed with an asterisk in each plot: *<0.05, **<0.01, and ***<0.001 (Pre, n = 16; Peri, n = 10; Post, n = 11).

**Figure 5:**
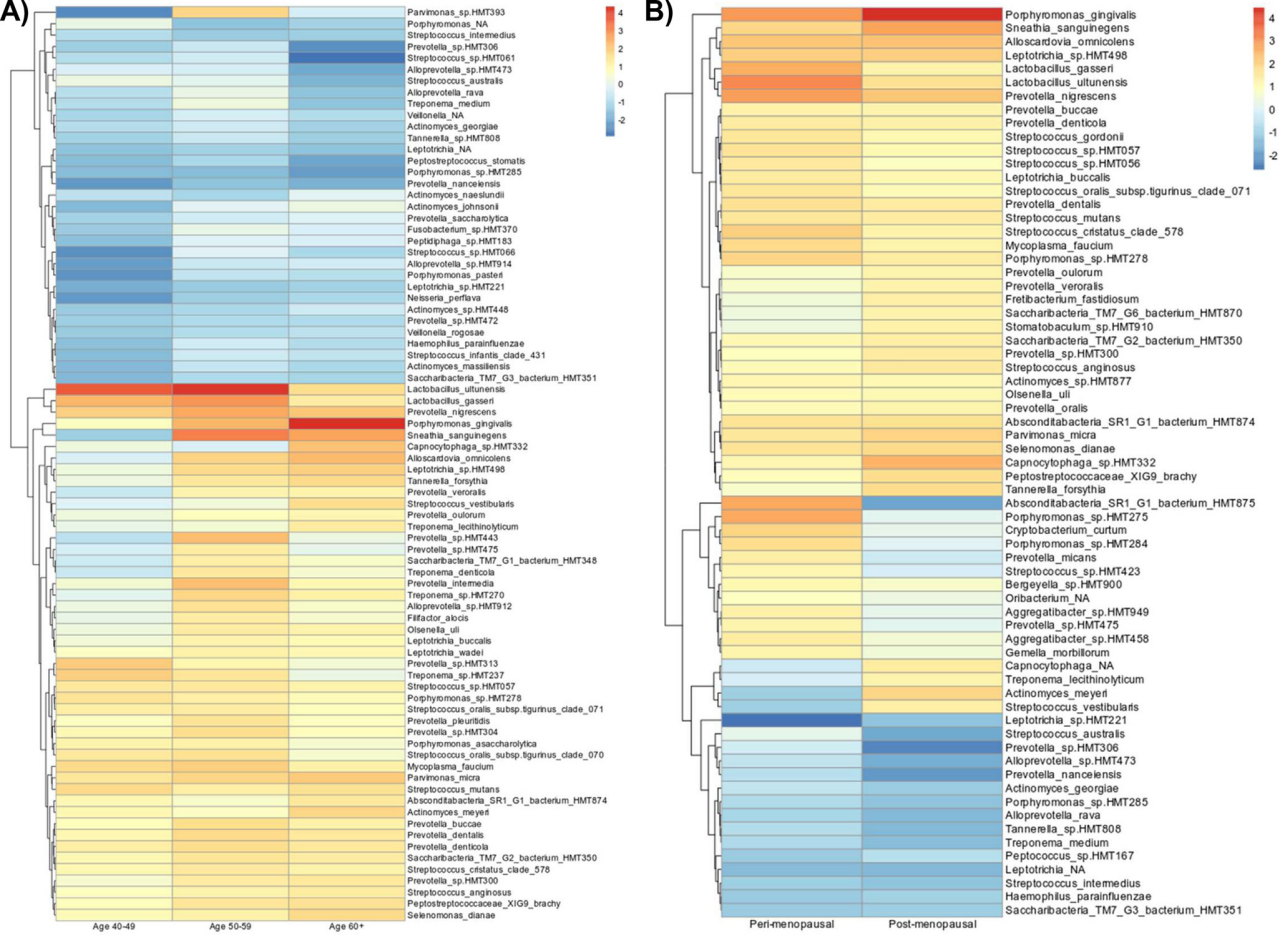
Differential abundance analysis in the women ageing and menopausal ageing groups. (A) Heatmap plot of log fold changes in microbial taxa abundance from ANCOM-BC analysis. Log fold change demonstrates the abundance when measured against the control group (age <32 years) (<32, n = 18; 40-49, n = 10; 50-59, n = 20; 60+, n = 12). (B) Heatmap plot of log fold changes in microbial taxa abundance from ANCOM-BC analysis. Log fold change demonstrates the abundance when measured against the control group (premenopausal) (Pre, n = 16; Peri, n = 10; Post, n = 11).

*Women ageing:* There were no significant differences between α-diversity (*P* > .05) and β-diversity (stress = 0.1936; *F* = 1.405; *P* = .072) between any of the female age groups (Figure 4). However, *Porphyromonas gingivalis* and *Tannerella forsythia* increased in abundance with female ageing; the highest abundance was seen in women aged >60 years (Figure 5). The ONRB species *Streptococcus oralis* and *Streptococcus australis* were reduced in abundance as women aged, with the lowest abundance seen in women aged >60 years. Conversely, the ONRB *Actinomyces massilensis* and *Actinomyces naeslundii* increased in abundance during ageing (Figure 5).

Thermodesulfobacteriota, not known to be nitrate reducing, was significantly different between the age groups (*P* = .002), with women aged <32 years having the lowest relative abundance (0.005% ± 0.014%) and women aged >60 years having the highest relative abundance (0.044% ± 0.128%) of this bacterial species.

*Menopausal ageing:* Similarly, there were no significant differences in α-diversity (*P* > .05) and β-diversity (stress = 0.2426; *F* = 1.349; *P* = .121) between the menopause groups (Figure 4). *P. gingivalis* and *T. forsythia*, as well as ONRB *Streptococcus oralis* and *Streptococcus mutans*, increased in abundance with menopausal ageing, with the highest abundance seen in the postmenopause group (Figure 5). Also, the ONRB *Actinomyces georgiae* and *Haemophilus parainfluenzae* were reduced in abundance in the peri- and postmenopause groups compared to the premenopause group (Figure 5). Again, Thermodesulfobacteriota was significantly different between the menopause groups (*P* = .046), with the premenopause group having the lowest relative abundance (0.005% ± 0.015%) and the postmenopausal group having the highest relative abundance (0.048% ± 0.134%).

## Discussion

In summary, this pilot study suggests that both the oral microbiome and microenvironment change during female ageing, with specific bacterial species being identified. Increasing age accompanies an increasing likelihood of being menopausal,^30^ but larger studies, including hormonal status, salivary flow, and full periodontal assessment, are needed to confirm this and fully evaluate the potential links proposed here between the menopausal status and specific bacteria that could affect oral and systemic health, as well as oral health management. The data produced here identify the outcome measures required for a larger and more robust study. They also provide preliminary data for power calculations for larger sample sizes in future studies investigating the oral microbiome during menopause. Further, rather than a retrospective analysis, collecting all the variables prospectively will also improve the robustness of this future work.

The increased systolic blood pressure and diastolic blood pressure reported with ageing and progressing menopause reflect the known heightened cardiovascular risk that has already been reported in older women.^6^^,^^42^ It is the decline in plasma oestrogen levels during menopause contributing to endothelial dysfunction, vascular stiffness, and impaired NO production^6^ that promotes this.^43^, ^44^, ^45^ Menopause is a consequence of aging, so the menopause-ageing-oestrogen interplay is highly related to vascular function; over 70% of age-related diseases are influenced by the effects of reproductive ageing.^5^ This overlap underscores the importance of examining menopause alongside measurement of changing oestrogen status in future studies, as oestrogen is a significant modifier of age-related health outcomes in women, which may include NO pathways.

Overall, our study did not demonstrate changes in the diversity of the oral microbiome during women’s ageing and increasing menopausal status, which agrees with Tramice et al.^14^ Nevertheless, subgroup analysis into smaller experimental groups could have affected the robustness of our oral microbiome data, with the lack of differences in α- and β-diversity suggesting a larger sample size than that provided in this pilot study, rather than a firm conclusion of unchanged microbial diversity at this time. However, looking at individual species, we observed that both *P. gingivitis* and *T. forsythia* significantly (*P* < .05) increased with female ageing and progressive menopausal status. *P. gingivalis* and *T. forsythia* are members of the red complex family, making them key periodontal pathogens known to have high inflammatory capacity, which perpetuates oral disease states.^46^ This “unbalancing” towards specific bacteria within the oral microbiome—namely, *P. gingivalis*, which is “causative” for periodontal disease—makes sense, as previous studies report increased periodontal disease (linked to *P. gingivalis*) with ageing,^47^ although the debate continues as to whether periodontitis is a natural consequence of getting older. One previous study supporting that oral microbiome changes may be secondary to hormonal changes rather than just “getting older” found that oestradiol, in a dose-dependent manner, increased *P. gingivalis* and its invasion of gingival epithelial cells *in vitro,* reporting thus that oestradiol could alter the virulence traits of *P. gingivalis.*^48^ Other *in vivo* studies have further associated declining oestrogen levels with periodontal disease, suggesting that HRT may aid oral health improvements.^49^^,^^50^ If this menopausal/hormonal link was proven with more *in vivo* research, it suggests that women may require personalised care pathways for rebalancing the oral microbiome and restoring oral health.

When specifically considering those bacteria involved in salivary nitrate-nitrite reduction, in our study, the abundance of some species of oral nitrate–reducing bacteria increased with both age (eg, *A. massilensis, A. naeslundii*) and menopausal status (eg, *S. oralis, S. mutans*), while others decreased in abundance with age (eg, *S. oralis, S. australis*) and progressing menopausal status (eg, *A. georgiae, H. parainfluenzae*). The inconsistent patterns in ONRB abundance across age and menopause status in our study are in agreement with our previous studies that ORNBs are more complex than first thought,^51^ and when investigating mechanisms, they must be considered in terms of different species-level *functionality* and overall activity.^14^^,^^52^, ^53^, ^54^ For this reason, the ONRA of bacteria was assessed in our study, with the ONRA being lowest in women aged <32 years and highest in women aged 50 to 59 and >60 years. This increase in ONRA (rather than the decrease we hypothesised), alongside the observed increase in systemic blood pressure, may reflect a compensatory physiological adaptation. Such a response has previously been reported in the gut microbiota, which can compensate for metabolic imbalance by influencing hormone release from enteroendocrine cells and regulating insulin sensitivity, glucose tolerance, fat storage, and appetite.^55^ Compensatory mechanisms in the oral cavity, however, are much less well understood and can remain only speculative at this time.

Here we also identify that, as well as the bacteria themselves, the oral microenvironment may change. Salivary buffering capacity was increased with female ageing and progressing menopausal status. Increased buffering capacity helps neutralise acids produced by bacteria,^56^ potentially protecting against dental caries and enamel erosion.^57^ However, buffering capacity does not necessarily control the growth of pathogenic bacteria such as red complex species (*P. gingivalis, T. forsythia*).^58^ The paradigm to address here, then, is that increased buffering capacity is usually related to increased salivary flow, yet menopause status is related to dry mouth in the literature.^11^^,^^56^ To unravel this, this pilot study suggests that larger future studies and menopause research must measure salivary flow alongside buffering capacity to understand how the changing oral microenvironment affects the oral microbiome. It is thus challenging to interpret changes to the oral microbiome and buffering capacity without also assessing flow.

This pilot study has several other limitations aside from the small sample size, which will be informative for future work. First, we opted to use BPE scores to assess periodontal health, as a full periodontal assessment was not part of the blood pressure screening protocols, and, retrospectively, BPEs were the most commonly used measurements by clinicians in routine care. We thus used BPE scores of 4 to identify the most extreme examples of participants with “potential periodontal concern.” It gave us some indication of those participants most likely to have periodontal disease, in agreement with the literature, as part of a pilot study. However, using this as a proxy in the absence of 6-point pocket charts may have led to underestimation or misrepresentation of periodontal disease. Thus, future studies should use a more detailed assessment of participants with potential periodontal concerns, including 6-point pocket charts and radiographs to diagnose periodontal disease more accurately.^40^

Another major limitation of this pilot study was that menopausal status was inferred from age rather than confirmed via clinical/hormonal data. Menopause is highly individual, and menopause status based only on age potentially introduces a misclassification bias. There was considerable correlation in most data obtained via age groups and menopausal status, which were then classified into menopausal groups based on the literature.^30^ Fortunately, there were similar patterns between ageing and menopausal groups in our study (blood pressure, salivary buffering capacity, oral microbiome abundance, and ONRA), but there were some subtle differences in the presence of oral nitrate–reducing species (ONRB) between women’s ageing and menopausal status, supporting this misclassification. Increasing chronological age was associated with reduced abundance of *S. oralis* and *S. australis*, with the lowest levels observed in women aged >60 years, while *A. massilensis* and *A. naeslundii* increased with age. In contrast, stratification by menopausal status demonstrated increased abundance of *S. oralis* in postmenopausal women, alongside reduced abundance of *A. georgiae* and *H. parainfluenzae* in peri- and postmenopausal groups compared to premenopausal women. This supports some of our earlier suggestions that *factors other than age are required for accurate classification of experimental groups* when undertaking a study of menopausal women.

Alongside verification of menstruation status in all ages of women, our suggestion, therefore, is that future larger studies on the oral microbiome should directly measure plasma oestrogen and follicle-stimulating hormone. We attempted to do this with salivary oestradiol status (Figure 1), as some studies suggest that salivary oestradiol should correlate with plasma levels, in which case, one would have expected changes in our study as women aged.^59^ However, the lack of oestradiol differences between age groups seen here suggests that *plasma* sampling may be essential, even if this is a more invasive approach. Without a clear hormonal definition, in this pilot study, there is a possible overlap of women experiencing menopause among the older and younger age groups, and some differences may have been missed. Future studies should further undertake more detailed medical histories and measure plasma hormones to improve the interpretation of oral microbiome changes in relation to menopause.

Finally, women using hormonal contraception and HRT were also included in this study, which may have influenced hormonal status and affected the oral microbiome outcomes. Thus, future studies with larger participant numbers will need to perform regression analysis or exclude this group to ensure that any form of hormone treatment is not modulating any of the oral microbiome changes seen here.

Despite these limitations, to our knowledge, this is the first study to highlight that women of menopausal age could have a changed oral microbiome that involves oral nitrate–reducing bacteria, also responsible for modulating blood pressure. These findings point to a complex, nonlinear relationship between age, hormone status, and microbial function, with implications for designing targeted interventions to modulate the oral microbiome that could also improve both oral and cardiovascular health. Findings also reinforce that oral and dental research should be conducted in both women and men, with awareness of the differences, as different hormonal factors are at play. As older women are known to be at increased risk of periodontal disease, dental caries, dry mouth, and burning mouth,^11^, ^12^, ^13^ going forward, we propose that women of menopausal age should receive enhanced oral hygiene, professional mechanical plaque removal, fluoride application, mouthwashes, artificial saliva substitutes, and HRT where appropriate—these approaches are currently recommended by clinicians to successfully prevent these symptoms.^60^ Oral hygiene measures should remain at the forefront, as this approach reduces *P. gingivalis* (causative for periodontal disease and increased with female ageing in this study), as well as creating a favourable microenvironment for healthy nitrate-reducing species. However, in addition, as we now understand that rebalancing the oral microbiome is key to oral health, novel treatments such as probiotics, mouth sprays, and dietary nitrate supplementation are future alternatives that need specific oral interventional studies to be performed in ageing and postmenopausal women.^29^^,^^61^ To date, probiotic studies during menopause have focused on the gut and vaginal microbiome but, unfortunately, have thus far neglected the mouth.^60^

In conclusion, our pilot observations suggest that the composition of the oral microbiome may change differentially at the species level as women age, alongside increasing blood pressure and worsening periodontal status; the latter being reported elsewhere, but causal links are yet to be established. The composition and activity of oral nitrate–reducing bacteria may also be altered during female ageing, but larger studies are required for confirmation, as these bacteria are linked to blood pressure. Our findings emphasise the critical need for both age- and sex-specific oral health care preventative strategies and treatments, plus further primary research studies in women that consider hormonal influences and microbiome dynamics so that the impact of menopause, and not just ageing, is fully understood. We propose that being a culturally competent clinician should include consideration of gender biology to better prevent and manage oral health, including periodontal disease and associated systemic conditions in female populations. Thus, more research is needed in women’s health and the oral microbiome to achieve this.

## Author contributions

K.J.M.: Lab analysis, data analysis, and wrote the manuscript. A.D.: Participant recruitment, sample collection, data analysis, lab analysis, and wrote the manuscript. A.S.S.: Data analysis, lab analysis, and project supervision. R.B.: Data analysis, lab analysis, and project supervision. C.S.I.: Conceived project and conducted literature review. T.L.N.: Participant recruitment and sample collection. S.H.: Conceived project. Z.L.S.B.: Conceived the project, project supervision, data analysis, wrote the manuscript, and acquired funding. All authors reviewed the manuscript and agreed on the published version.

## Conflict of interest

The authors declare that they have no known competing financial interests or personal relationships that could have appeared to influence the work reported in this paper.
